# Molecular Epidemiology of Antibiotic-Resistant *Escherichia coli* from Farm-to-Fork in Intensive Poultry Production in KwaZulu-Natal, South Africa

**DOI:** 10.3390/antibiotics9120850

**Published:** 2020-11-27

**Authors:** Katherine S. McIver, Daniel Gyamfi Amoako, Akebe Luther King Abia, Linda A. Bester, Hafizah Y. Chenia, Sabiha Y. Essack

**Affiliations:** 1 Antimicrobial Research Unit, College of Health Sciences, University of KwaZulu-Natal, Durban 4000, South Africa; katherine.mciver@gmail.com (K.S.M.); amoakodg@gmail.com (D.G.A.); essacks@ukzn.ac.za (S.Y.E.); 2 Biomedical Resource Unit, School of Laboratory Medicine and Medical Sciences, College of Health Sciences, University of KwaZulu-Natal, Durban 4000, South Africa; 3 Discipline of Microbiology, School of Life Sciences, College of Agriculture, Engineering and Sciences, University of KwaZulu-Natal, Durban 4000, South Africa; Cheniah@ukzn.ac.za

**Keywords:** antibiotic resistance, antibiotic resistance genes, *Escherichia coli*, intensive poultry production, farm-to-fork continuum, South Africa

## Abstract

The increased use of antibiotics in food animals has resulted in the selection of drug-resistant bacteria across the farm-to-fork continuum. This study aimed to investigate the molecular epidemiology of antibiotic-resistant *Escherichia coli* from intensively produced poultry in the uMgungundlovu District, KwaZulu-Natal, South Africa. Samples were collected weekly between August and September 2017 from hatching to final retail products. *E. coli* was isolated on eosin methylene blue agar, identified biochemically, and confirmed using polymerase chain reaction (PCR). Susceptibility to 19 antibiotics was ascertained by the Kirby–Bauer disc diffusion method. PCR was used to test for resistance genes. The clonal similarity was investigated using enterobacterial repetitive intergenic consensus polymerase chain reaction (ERIC-PCR). In total, 266 *E. coli* isolates were obtained from all the samples, with 67.3% being non-susceptible to at least one antibiotic tested and 6.7% multidrug resistant. The highest non-susceptibility was to ampicillin (48.1%) and the lowest non-susceptibility to ceftriaxone and azithromycin (0.8%). Significant non-susceptibility was observed to tetracycline (27.4%), nalidixic acid (20.3%), trimethoprim-sulfamethoxazole (13.9%), and chloramphenicol (11.7%) which have homologues used in the poultry industry. The most frequently observed resistance genes were *bla_CTX-M_* (100%), *sul1* (80%), *tetA* (77%), and *tetB* (71%). ERIC-PCR grouped isolates into 27 clusters suggesting the spread of diverse clones across the farm-to-fork continuum. This reiterates the role of intensive poultry farming as a reservoir and a potential vehicle for the transmission of antibiotic resistance, with potentially severe public health implications, thus, requiring prompt and careful mitigation measures to protect human and environmental health.

## 1. Introduction

Poultry farmers have been using antibiotics since the 1940s when it was discovered that the application of antibiotics at subtherapeutic levels resulted in better feed conversion and reduced bird mortality [1]. Antibiotics reduced diseases such as necrotic enteritis and colibacillosis [2,3] as well as the incidence of human illness from chicken products such as enteritis caused by *Salmonella*, *Campylobacter,* or pathogenic strains of *Escherichia coli* [2,4]. However, the use of these therapeutic agents in the poultry industry has increased drastically in recent years due to an increased demand for animal protein, which has led to an intensification of poultry farming to provide for the demand [5].

Intensive farming involves much higher stocking densities than extensive or subsistence farming, resulting in close contact between animals and increased stress levels [6]. These factors lower the immunity of the animals, predisposing them to disease development and the spread of disease due to close contact [7]. Measures such as good husbandry practices, good biosecurity and immunization programs help to prevent disease outbreaks in flocks [8]. Where these measures are not in place, antibiotics are used to compensate and maintain production [9]. Despite the beneficial effects of antibiotic use in ensuring the animals are healthy, and meat production is optimized, one major drawback is the development of resistance to these antibiotics by bacteria. This has resulted in intensive poultry farms becoming potential reservoirs of antibiotic-resistant bacteria (ARB), and antibiotic resistance genes (ARGs) that can be transferred to humans through the food chain [10].

Antibiotic resistance has become among the top threats to humans globally, with devastating public health consequences and enormous economic loss [11]. To adequately address this problem, it is imperative to understand the extent to which humans, animals and the environment contribute to the spread of ARB and ARGs. However, many studies conducted in this regard, specifically in food-producing animals, have focused on a single point such as the farm [12], abattoir [13], or the retail point [14].

Although necessary, in their respect, such studies did not provide a full picture of the entire production chain. To achieve such a holistic picture, a farm-to-fork approach that takes into consideration all the critical points from the farm to the final packaging is necessary. In addition, isolation of specific pathogens, such as *Campylobacter*, could be hindered by the complexity and the unique requirements for their laboratory isolation. However, some organisms, such as *Escherichia coli*, for which the mechanism of antimicrobial resistance are well understood, are considered to be suitable indicator organisms [15]. *E. coli*, particularly Avian Pathogenic *E. coli* (APEC), causes colibacillosis in poultry, which is a significant cause of economic losses in broiler and layer production [16]. The organism is ubiquitous and can be transferred among humans, animals, and the environment and has been shown to transfer plasmids and other mobile genetic elements easily [17]. Therefore, *E. coli* is a good indicator bacterium in antibiotic resistance surveillance in poultry. Thus, the current study aimed to delineate the molecular epidemiology of antibiotic-resistant *E. coli* from farm-to-fork in an intensive poultry production system in the uMgungundlovu District, KwaZulu-Natal, South Africa. The results of such a study would provide intensive poultry farmers and the public health sector with information necessary to develop mitigating measures against the spread of antimicrobial resistance in the poultry industry and to humans.

## 2. Results

### 2.1. Enumeration of Escherichia coli

**Figure 1:**
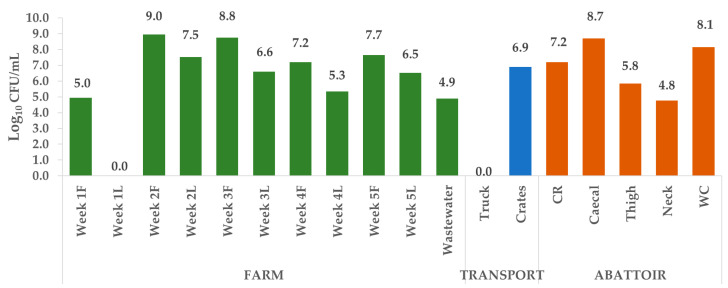
Quantification of *Escherichia coli* per sampling point. For the farm samples (Week 1–5), F, feces and L, litter. At the abattoir, CR, carcass rinsate and WC, whole chicken.

Three hundred and forty-five (345) putative *E. coli* isolates were cultured from a total of 162 samples collected from the different sources along the continuum over the five weeks (samples from the farm, transport, and abattoir). Of these, 266 (77%) were confirmed as *E. coli* by biochemical analyses and real-time PCR (Table A1, Appendix A). No isolates were cultured from the Week 1 litter and truck samples. For ease of presentation, the colony forming units per mL (CFU/mL) of sample were log_10_ transformed. The abundance of *E. coli* varied per sampling point, with the farm (Week 2) recording the overall highest number of CFU/mL (9.0) and cecal samples having the highest at the abattoir (8.7) (Figure 1).

### 2.2. Antibiotic Susceptibility

#### 2.2.1. Overall Susceptibility Profile

**Figure 2:**
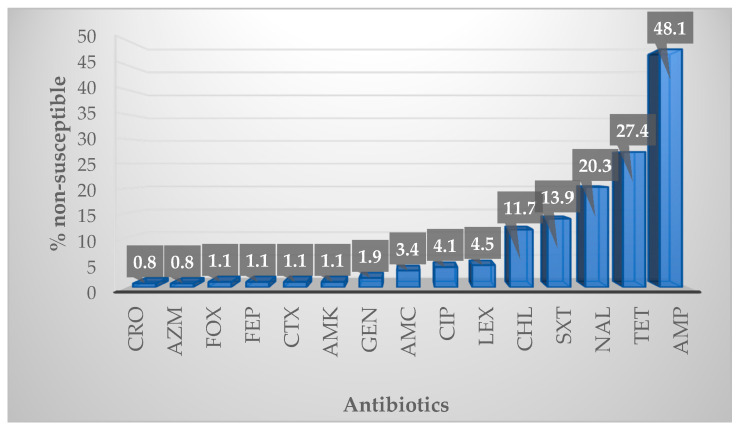
Percentage non-susceptibility of all isolates to the antibiotics tested. CRO, ceftriaxone; AZM, azithromycin; FOX, cefoxitin; FEP, cefepime; CTX, cefotaxime; AMK, amikacin; GEN, gentamicin; AMC, amoxycillin-clavulanic acid; CIP, ciprofloxacin; LEX, cefalexin; CHL, chloramphenicol; SXT, trimethoprim-sulfamethoxazole; NAL, nalidixic acid; TET, tetracycline; AMP, ampicillin.

One hundred and sixty-seven (67.3%) of the 266 isolates tested showed non-susceptibility to at least one of the antibiotics included in this study (Figure 2). The highest non-susceptibility was observed to ampicillin (48.1%), while the lowest was to ceftriaxone (0.8%) and azithromycin (0.8%). Significant non-susceptibility was also observed to tetracycline (27.4%), nalidixic acid (20.3%), trimethoprim-sulfamethoxazole (13.9%), and chloramphenicol (11.7%). All isolates tested were 100% susceptible to ceftazidime, imipenem, meropenem and tigecycline.

**Figure 3:**
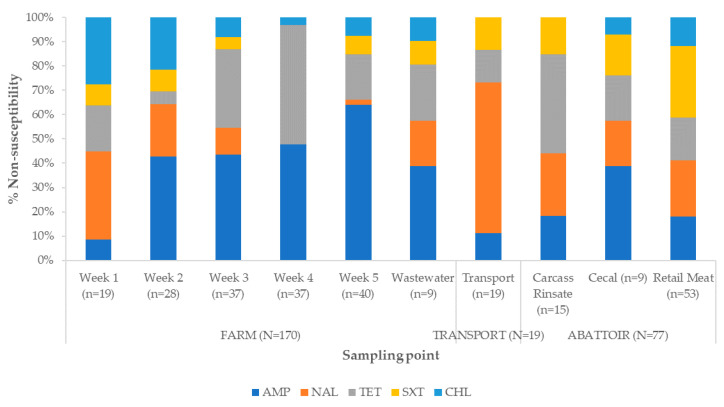
Percentage non-susceptibility of isolates to selected antibiotics stratified by sample source. AMP, ampicillin; NAL, nalidixic acid; TET, tetracycline; SXT, trimethoprim-sulfamethoxazole; CHL, chloramphenicol.

There was an increased percentage of ampicillin-resistant isolates in Week 2 and Week 5 as well as in the carcass rinsate and wastewater samples (Figure 3). A similar trend was observed in nalidixic acid. High percentages of tetracycline-resistant isolates were recorded in Weeks 3 to 5 and the highest percentage was in cecal samples. For trimethoprim-sulfamethoxazole, the highest percentage of resistant isolates was observed in Week 2, cecal and wastewater samples, while for chloramphenicol this was recorded in Week 2 and wastewater. The frequency of resistance to chloramphenicol was lower for the remaining samples. Overall, the percentage of resistant isolates in retail meat was lower for all antibiotics as compared with that in the fecal, carcass rinsate, cecal, and wastewater samples.

#### 2.2.2. Antibiotic Susceptibility Profiles and Multidrug Resistance Patterns

Grouping of the non-susceptible isolates yielded 33 different antibiotic susceptibility profiles (Table 1). The largest group was made up of isolates with non-susceptibility to a single antibiotic (119), with the largest proportion being non-susceptibility to ampicillin alone (56). In addition, 17 (6.7%) isolates were non-susceptible to antibiotics in three or more different antibiotic classes and were classified as multidrug resistant (MDR) (Table 1). Among these MDR isolates, the highest percentage was recorded in the farm (wastewater) samples, while none was recorded in transport samples. Interestingly, retail meat samples (abattoir) also recorded 9.4% MDR isolates (Table S1 in Supplementary Materials).

#### 2.2.3. Detection of Antibiotic Resistance Genes

Apart from the quinolone resistance gene, *qnrS*, all the genes tested in the current study were amplified in at least one of the studied isolates (Table 2). The predominant resistance genes were the *bla_CTX-M_* (100%), *sul1* (80%), *tetA* (77%), and *tetB* (71%).

### 2.3. Determination of Clonality

**Figure 4:**
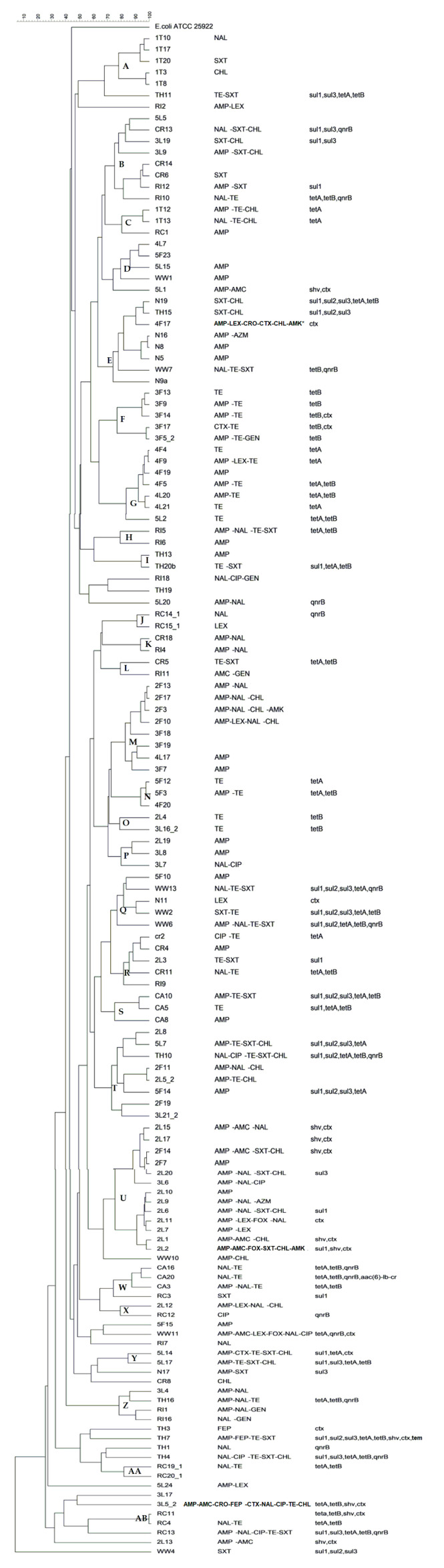
Dendrogram showing the clonal relationship among isolates with antibiotic susceptibility profiles and resistance genes.

Isolates could be divided into 27 clusters based on a 75% fingerprint similarity, with several isolates sharing greater than 90% similarity (Figure 4). Among the 27 clusters, eight contained isolates from the same sample, and two of these clusters contained five isolates each. Cluster A contained isolates from Week 1 feces from the hatchling trays, all of which displayed different antibiotic susceptibility profiles. Cluster F were all from Week 3 fecal samples but as compared with cluster A there were some similarities in susceptibility profiles, with all isolates being non-susceptible to tetracycline and three to ampicillin.

The largest cluster had thirteen isolates from three samples (that is, Week 2 litter with ten isolates, Week 2 feces with two isolates, and Week 3 litter with one isolate). Three isolates from Week 2 litter and one from Week 2 feces had the same antibiotic susceptibility profile, AMP-NAL-SXT-CHL. All the isolates were non-susceptible to ampicillin. The rest of the antibiotic susceptibility profiles varied.

There were only a few instances in which isolates from different samples demonstrated the same antibiotic susceptibility profiles and appeared in the same cluster. One cluster had a crate sample CR6 which showed greater than 90% similarity to a carcass rinsate sample from the abattoir RI12, and both had the same antibiotic susceptibility profile, AMP-SXT. In another cluster, both a litter sample from Week 2 and litter sample from Week 3 showed resistance to tetracycline. Cluster P also had a Week 2 litter and a Week 3 litter sample with ampicillin non-susceptibility and >85% similarity.

## 3. Discussion

This study investigated antibiotic resistance in *Escherichia coli*, from hatching to the final retail product, over six weeks in a flock of intensively produced chickens in the uMgungundlovu District, KwaZulu-Natal, South Africa. *E. coli* was isolated at all stages of the farm-to-fork continuum (farm, transport, and abattoir). Over 60% of the isolates obtained across all sampling points showed resistance to at least one of the antibiotics tested. Multidrug-resistant isolates were also recorded along the continuum and isolates harbored genes that confer resistance to tetracycline, quinolones, and extended-spectrum β-lactams. Although the clonality did not show a likelihood of relatedness between the isolates from the different sources, resistance to most antibiotics was observed at almost every sampling point, thus demonstrating the role of intensive poultry farming as a significant reservoir and potential vehicle for the transmission of antibiotic-resistant bacteria to humans and the environment.

### 3.1. Enumeration of Escherichia coli

*Escherichia coli* is a normal flora in the intestine of humans and animals; thus, it has been used as a suitable indicator of fecal pollution in different matrices. This explains the high *E. coli* counts observed in the fecal and cecal samples in the present study. However, other strains of this organism have evolved to cause infections in humans [18] and animals, including chickens [19]. In addition, Ewers et al. [20] reported that *E. coli* isolated from chicken intestine had zoonotic potentials. This meant that farm workers needed to observe strict personal protective measures within the farms, especially during the cleaning of farmhouses that could contain highly contaminated fecal matter, as recorded in the current study. More worrisome was the fact that retail portions (neck and thigh) and whole chicken samples at the packaging stage harbored up to 10^8^ *E. coli* counts. This high count could be due to cross-contamination between the chicken intestine, carcass, the environment, and meat portions, as described in the poultry-processing microbial risk assessment model proposed by Nauta and colleagues [21]. Although not tested in the present study, this high *E. coli* count may include pathogenic strains that could cause infection to consumers if the meat is not adequately cooked. However, such risk would need to be determined using appropriate risk assessment tools [22], and improved hygienic conditions should be implemented within abattoirs.

### 3.2. Antibiotic Susceptibility

The World Health Organization (WHO) has recommended the use of *E. coli*, among other bacteria, as a priority organism for the global surveillance of antimicrobial resistance [15]. The present study tested the susceptibility of 266 *E. coli* isolates to 19 antibiotics and recorded a 67.29% non-susceptibility to at least one of the tested antibiotics. This overall percentage non-susceptibility was, however, lower than that recorded in two other studies on *E. coli* from poultry in South Africa [19,23]. The differences in susceptibility between the current study and the previous ones could be because of several factors such as the study design, whether the studied birds were antibiotic fed, the antibiotic panel used, the number of isolates tested, and the susceptibility testing method used. Neither of these studies used the farm-to-fork testing protocol but focused on a single sampling point along the continuum. For example, Theobald et al. examined cecal samples at processing, and these were from chickens that had received continuous antibiotic treatment [23]. Similarly, Oguttu et al. only analyzed specific *E. coli* strains that were isolated from sick chickens (colibacillosis samples) [19]. Again, colibacillosis samples would be expected to have higher frequencies of resistance due to increased incidence of therapeutic antibiotic use, that is, for the treatment of clinically ill birds [24]. Therefore, the low antibiotic resistance, in this study, could be due to reduced use of therapeutic antibiotics as the birds were mostly healthy, and good biosecurity. The flock in the current study received growth promoters in feed, consisting of salinomycin and zinc bacitracin (ZB). Both compounds were incorporated into the starter, grower, and finisher feed, while no ionophores nor antibiotics were present in the post-finisher feed. According to farm management, no therapeutic antibiotics were used in this flock. However, although it has been reported that *E. coli* is intrinsically resistant to ZB, other components of the feed, like zinc, have been shown to co-select for antibiotic resistance to tetracycline, chloramphenicol, and beta-lactams [25], and thus could have influenced the resistance recorded in the current study.

While some microorganisms show resistance to single antibiotics, some others have developed complex mechanisms allowing them to resist treatment with antibiotics from different classes. This has prompted international bodies such as the WHO and Centers for Disease Control to call for a global war against multidrug-resistant microorganism [26]. In the current study, MDR *E. coli* isolates were isolated in the farm, and abattoir but not in the transport samples (Table S1). There was an increase in the percentages of multidrug-resistant isolates over the farm-to-fork continuum with the highest percentage recorded in the wastewater (farm) and cecal (abattoir) samples, followed by a decrease in retail meats samples. The higher MDR percentage observed in the wastewater samples was not surprising, as the isolates would have been exposed to the poultry environment for longer periods and more environmental stressors, despite the withdrawal of the feed additives at some stages before slaughter. Additionally, although some studies have reported that salinomycin was safe as a feed additive [27], a study in Canada revealed that *E. coli* isolated from chickens fed with salinomycin-supplemented feed showed MDR. It was suggested that there could be an association between its use and the co-selection of several resistance genes for tetracycline, sulfonamides, chloramphenicol, and aminoglycosides, all of which are often found on class 1 integrons [28]. However, the detection of MDR in the retail meat samples, even though at a relatively low percentage, calls for the institution of measures to prevent the spread of such bacteria to humans through the food chain.

The overall higher non-susceptibility and MDR recorded in the wastewater and cecal samples were matched by the presence of resistance genes, with percentages of most of the genes being higher in cecal and wastewater samples (Table 2). Seventy-four percent of isolates tested positive for at least one of the resistance genes investigated in this study. However, resistance could not be attributed to these genes in all isolates and may have been caused by other mechanisms that were not investigated in the current study. The majority of tetracycline non-susceptible isolates contained either *tet*A or *tet*B genes, similar to other reported studies [29,30,31]. It has also been reported that other factors, such as ribosomal protective proteins (RPP) or mono-oxygenase enzymes, could inactivate tetracycline [32]. These could explain the tetracycline non-susceptibility observed in the remaining isolates that harbored neither of the *tet* genes investigated.

Resistance to sulfonamides, quinolones, and extended-spectrum cephalosporins is also a major concern globally [15]. In the present study, the *sul3* gene was more detected than the *sul2*, contrary to previous studies that have reported an opposite trend, i.e., the *sul1, sul2* and *sul3* encode resistance to sulfamethoxazole [29,30,33]. Zinc bacitracin has also been associated with an increase in the prevalence of the *sul1* and *sul2* resistance genes, which were found in this study [28]. A few mobile quinolone resistance genes were also detected in this study, notably the 16 *qnrB* and one *aac(6)-lb-cr* gene. The other mechanism of resistance for quinolones is mutations to DNA gyrase in Gram-negative bacteria and an increase in efflux or porin loss [34,35]. These were not, however, tested in the present study. Furthermore, all isolates with third and fourth generation cephalosporin non-susceptibility were screened for *bla_TEM_, bla_CTX-M_*, and *bla_SHV_* genes. All isolates tested contained *bla_CTX-M,_* which was higher than other reported rates [36,37]. Among these, 4/14 were found in retail meat samples, and therefore could indicate a public health risk of transfer to humans via the food chain.

### 3.3. Determination of Clonality

There was no evidence of the dissemination of bacterial clones across the farm-to-fork continuum, as evident from the diversity in clusters, antibiotic susceptibility profiles, and resistance genes. Instead, there appeared to be a de novo emergence of resistance traits at different time points, possibly through the loss or acquisition of mobile genetic elements throughout the study period, from hatching to final processing. Similar findings were seen in a study of *E. coli* from different stages of processing of chicken carcasses conducted by Geornaras and Hastings (2001), in which clonality (amplified fragment length polymorphism) did not correspond to plasmid profiles nor antibiotic susceptibility profiles [38]. Focusing on the resistance patterns of the isolates, the diverse clonality shown by the enterobacterial repetitive intergenic consensus polymerase chain reaction (ERIC-PCR) results suggests that (1) the same clones might not have been transmitted across the farm-to-fork continuum; (2) isolates might have lost or gained resistance genes or plasmids over time across the production line; and (3) external factors such as birds from different farmhouses processed by the same abattoir, might not be ignored.

Although it is generally accepted that the use of antibiotics promotes the selection for antibiotic-resistant isolates [9,10,39,40], other factors not investigated in the current study could have influenced the recorded non-susceptibility. For example, resistance may also be introduced from the environment, with hatchlings’ guts being colonized within a few hours of hatching [41,42]. This could lead to horizontal gene transfer between bacteria in the hatchery or house and bacteria in the chickens’ guts [42]. In addition, the studied farm operated an all-in/all-out system in which flocks from one house were not mixed with others but were, instead, all sent to the slaughter at the same time. This was followed by complete cleaning and disinfection of the whole house before the introduction of a new flock. Thus, if not properly disinfected, some bacteria may survive and serve as a source of resistance to the next flock entering [23]. This could be assumed in the current study given that the highest percentage of MDR isolates and resistance genes were from wastewater after cleaning of the house. Biosecurity is another source of resistance; flies and rodents have been known to carry resistant organisms, which can be transferred to poultry if houses are not sealed. The same applies to workers in the house. Furthermore, the observed resistance profiles could be due to the historical use of antibiotics such as doxycycline, sulfadiazine, trimethoprim, enrofloxacin, olaquindox, avilamycin, tylosin, and kitasamycin tartrate, in the poultry industry.

Therefore, it is suggested that future studies investigate these aspects to determine the extent of their involvement in the detection of non-susceptible bacteria in poultry and poultry environments. Nevertheless, the observed non-susceptibility to clinically relevant antibiotics, including those mentioned in the WHO priority list, is an alert to the potential human health hazards associated with poultry and requires adequate attention.

## 4. Materials and Methods

### 4.1. Ethical Considerations

This study formed part of a larger project for which ethical clearance had been obtained from the Animal Research Ethics Committee (reference AREC 073/016PD) and the Biomedical Research Ethics Committee (reference BCA444/16) of the University of KwaZulu-Natal. The study was also placed on record with the South African National Department of Agriculture, Forestry, and Fisheries (reference 12/11/1/5 (879)).

### 4.2. Study Population, Sampling, and Sample Processing

The sampling protocol recommended by the World Health Organization Advisory Group on the Integrated Surveillance of Antimicrobial Resistance (AGISAR) was used. A detailed description of the sampling site, the sampling protocol, and sample processing have previously been described [43,44]. Briefly, one batch of one-day-old Cobb chickens was followed from hatching, in a single farm in the uMgungundlovu District of South Africa, to slaughter and final retail product, using a farm-to-fork methodology. The sampling took place in August and September of 2017. Sampling points included the farm (litter, feces, and wastewater collected on the last week from washing the birds’ house), transport (truck and crates); abattoir (carcass rinsate, cecal, neck and thigh portions, and whole chickens). Samples from each sampling round were transported to the laboratory on ice and analyzed within 6 h from the collection.

### 4.3. Bacterial Isolation, Purification, and Identification 

Following processing, 100 µL of each sample dilution was pour-plated onto eosin methylene blue (EMB) agar (Sigma-Aldrich, St. Louis, MO, USA) and incubated for 18 to 24 h at 37 °C. Colonies with characteristic green metallic sheen were counted and recorded as colony forming units per mL (CFU/mL) of the processed sample.

Then, twenty presumptive *E. coli* isolates were selected from each EMB plate and subcultured onto sorbitol MacConkey agar with 5-bromo-4-chloro-3-indolyl beta-d-glucuronide (BCIG) (Oxoid, Basingstoke, UK) to obtain pure colonies. Plates were incubated at 37 °C for 18 to 24 h. A single colony that displayed a blue-purple appearance, typical of *E. coli*, was selected from each plate, subcultured further on Nutrient Agar (Neogen, Lansing, MI, USA) and incubated for a further 18 to 24 h at 37 °C.

Following incubation on nutrient agar, isolates were subjected to a battery of biochemical tests. The isolates were first subjected to the string test [45], and those that were Gram-negative were then tested for oxidase and catalase production [46]. Oxidase-negative and catalase-positive isolates were then inoculated into triple sugar iron (TSI) agar slants. Isolates with typical TSI slant results (acid in base and on slant with gas production) were presumed to be *E. coli* [47] and stored in 10% glycerol TSB (tryptone soya broth; Oxoid) solution at −80 °C until needed for further testing.

### 4.4. DNA Extraction and Molecular Confirmation of Isolates

Colonies were suspended in 300 µL of distilled water and boiled for 20 min. Then, samples were placed on ice for 5 min before being centrifuged at 13,000 rpm for 3 min. The supernatant was extracted and used as template DNA for molecular confirmation of the isolates using real-time polymerase chain reaction (PCR) targeting the *uidA* gene. All primers were purchased from Inqaba Biotechnical Industries (Pty) Ltd. (Pretoria, South Africa). The primer sequences and controls used are shown in Table A2 (Appendix A).

PCR was conducted on a QuantStudio 5 Real-Time PCR System (Thermo Fischer Scientific, Johannesburg, South Africa). All reactions were conducted in a total volume of 10 uL which consisted of 5 µL of PowerUp^TM^ SYBR^®^ Green Master Mix (Thermo Fisher Scientific, Johannesburg, South Africa) 0.5 µL of the forward and reverse primers at a final concentration of 0.5 mM, 1 µL of molecular grade nuclease-free water, and 3 µL of template DNA. Cycling conditions included an initial Uracil-DNA glycosylase (UDG) activation at 50 °C for 2 min and Dual-Lock^TM^ polymerase activation at 95 °C for 2 min. This was followed by 35 cycles of denaturation (95 °C for 15 s), annealing (60 °C for 15 s), and extension (72 °C for 10 s). A final extension was achieved at 72 °C for 5 min [48]. A high-resolution melting curve analysis was performed by ramping up the temperature from 65 °C to 95 °C on a continuous mode [43]. *E. coli* ATCC 25922 was used as a positive control, and nuclease-free water was used as no template control.

### 4.5. Antibiotics Susceptibility Testing (AST)

Antibiotic sensitivity testing was done using the Kirby–Bauer disc diffusion assay according to Clinic and Laboratory Standards Institutes (CLSI) guidelines, and isolates were categorized as susceptible or non-susceptible using CLSI or the European Committee on Antimicrobial Susceptibility Testing (EUCAST) clinical breakpoints, depending on the disc concentration [49,50]. The following antibiotics were tested: ampicillin 10 µg, cefepime 30 µg, cefotaxime 30 µg, ceftazidime 30 µg, cefoxitin 30 µg, cefalexin 30 µg (EUCAST), gentamicin 10 µg, imipenem 10 µg, meropenem 10 µg, nalidixic acid 30 µg, ciprofloxacin 5 µg, tigecycline 15 µg (EUCAST), amoxycillin-clavulanic acid 20 µg + 10 µg, amikacin 30 µg, chloramphenicol 30 µg, azithromycin 15 µg, tetracycline 30 µg, trimethoprim-sulfamethoxazole 1.25 µg + 23.75 µg, ceftriaxone 30 µg, based on the WHO AGISAR recommended list [51]. All antibiotics were purchased from Thermo Fisher, Johannesburg (Oxoid) and Davies Diagnostics (Pty) Ltd. (Mast).

### 4.6. Antibiotic Resistance Genes Detection

According to the phenotypic resistance displayed by each isolate after the AST, a total of 120 isolates that showed resistance to at least one antibiotic in the tetracyclines, quinolones, sulfonamides, or cephalosporin classes were selected and tested further for the presence of genes conferring resistance to these antibiotics. Real-time PCR was used to detect the following genes: *tetA, tetB, qnrB, qnrS, aac(6)-lb-cr, sul1, sul2, sul3, bla_SHV_,* and *bla_CTX-M_,* except for the *bla_TEM_* gene.

PCR conditions included a UDG activation at 98 °C for 50 s. This was followed by 30 cycles of denaturation (95 °C for 10 s), annealing (62 °C for 30 s), and extension (72 °C for 20 s). A final extension was achieved at 72 °C for 5 min. Then, a melting curve was generated, as mentioned previously for the molecular confirmation of isolates.

Conventional PCR, on a BIO-RAD T100 Thermal Cycler (Bio-Rad, Johannesburg, South Africa) was used to test for the *bla_TEM_* resistance gene. Conditions were as follows: an initial activation of 94 °C for 3 min followed by 34 cycles of denaturation (94 °C for 1 min), annealing (55 °C for 1 min), and extension (72 °C for 1 min and 30 s). A final extension was achieved at 72 °C for 7 min [48].

PCR products from conventional PCR were subject to electrophoresis in a 1% agarose gel containing 5 µL ethidium bromide at 100V for 45 min in a 1X Tris-Acetate EDTA (TAE) buffer. A New England Biolabs Quick-Load^TM^ 1 kb ladder Quick-load (Inqaba Biotechnical Industries (Pty) Ltd., Pretoria, South Africa) was used as the standard. Gels were visualized using the Gel Doc™ XR+ imaging system (Bio-Rad, Johannesburg, South Africa). The product size was 980 bp.

### 4.7. Determination of Clonality

Representative isolates were selected for the clonality experiment based on antibiotic susceptibility profiles, where isolates from different sources belonging to the same antibiotic susceptibility profile were subjected to ERIC-PCR. A total of 138 isolates were selected from samples across the farm-to-fork continuum, and DNA was extracted using the GeneJET Genomic DNA purification kit (ThermoFisher Scientific, Johanessburg, South Africa) following the manufacturer’s guidelines. The PCR mixture was made up of 12.5 µL of DreamTaq Green PCR Master Mix (2X) (ThermoFisher Scientific, Johanessburg, South Africa), 0.1 µL of each forward and reverse primers (final concentration 1 µM), 3.3 µL of nuclease-free water and 4 µL of template DNA in a final reaction volume of 20 µL. PCR was run on Bio-Rad T100^TM^ Thermal Cycler (Bio-Rad, Johanessburg, South Africa) with the following conditions: initial denaturing at 95 °C for 2 min followed by 34 cycles of denaturation (90 °C for 30 s), annealing (52 °C for 1 min) and extension (65 °C for 8 min) and a final extension of 65 °C for 16 min. PCR products were subjected to electrophoresis in a 1% agarose gel at 75V for 150 min in a 1X Tris-Acetate EDTA (TAE) buffer. A New England Biolabs Quick-Load^TM^ 1 kb ladder Quick-load (Inqaba Biotechnical Industries (Pty) Ltd., Pretoria, South Africa) was used as the standard. Gels were stained in ethidium bromide solution for 15 min, destained for 10 to 30 min in distilled water, and visualized using a Gel Doc™ XR+ imaging system (Bio-Rad, Johannesburg, South Africa). Captured gel images were analyzed using the BioNumerics software version 6.6 (Applied Maths NV, Sint-Martens-Latem, Belgium). A band tolerance of 10% was used for inputting gel images. Cluster generation used Pearson correlation with a 1% optimization and an unweighted pair group with arithmetic averages (UPGMA) to create a dendrogram. Clusters were determined using a 75% similarity cut-off.

All primer, primer sequences, and controls used for the detection of AGRs and clonality are shown in Table A2 (Appendix A).

## 5. Conclusions

The present study revealed the abundance of *E. coli* at all points of the farm-to-fork continuum, with an overall 67.29% non-susceptibility. Multidrug resistance was also recorded at the farm and abattoir. As the only antibiotics used in the studied flock were growth promoters, it is postulated that co-selection of resistance genes and horizontal gene transfer may have contributed to this observed non-susceptibility. Non-susceptible isolates harbored one or more resistance genes, although these genes could not fully explain the overall non-susceptibility observed, suggesting that other factors not investigated in the current study could have also contributed. The clonality analysis through the ERIC-PCR also revealed a high diversity of clones across the farm-to-fork continuum, suggesting that the isolates might have acquired de novo resistance genes or lost some, especially plasmid-borne ones, over time. Nevertheless, the observed non-susceptibility to clinically relevant antibiotics, including those mentioned in the WHO priority list, is an alert to the potential human health hazards associated with poultry and requires adequate attention. It should, however, be noted that the current study only involved a single flock in a single farm, and results should not be over-generalized. Therefore, further studies that consider more flocks from many farms, the farmworkers, the poultry environment (including flies and rodents), and utilizing advanced tools such as whole-genome sequencing, could provide an in-depth understanding of the molecular epidemiology of antibiotic resistance in intensive poultry farming.

## Figures and Tables

**Table 1:** Number of isolates per antibiotic susceptibility profile stratified by source.

Antibiotic Susceptibility Profiles	Farm (*n* = 170)	Transport (*n* = 19)	Abattoir (*n* = 77)	Total (*n* = 266)
Susceptible	50	9	28	87
AMP	47	1	8	56
AZM	1	0	1	2
CHL	1	1	0	2
CIP	0	0	1	1
GEN	0	0	2	2
LEX	2	0	2	4
NAL	2	0	7	9
SXT	2	1	2	5
TET	33	4	1	38
AMP-AMK	1	0	0	1
AMP-CHL	3	0	0	3
AMP-LEX	2	0	1	3
AMP-NAL	2	1	0	3
AMP-SXT	0	0	1	1
AMP-TET	1	0	0	1
LEX-TET	1	0	0	1
NAL-TET	0	1	9	10
SXT-CHL	6	1	2	9
TET-CHL	1	0	0	1
TET-GEN	1	0	0	1
TET-SXT	1	0	4	5
AMP-LEX-CHL	1	0	0	1
AMP-NAL-GEN	0	0	1	1
AMP-NAL-TET	0	0	2	2
AMP-TET-SXT	1	0	2	3
NAL-TET-SXT	3	0	2	5
AMP-TET-SXT-CHL	2	0	0	2
NAL-CIP-TET-SXT	0	0	1	1
AMP-CTX-TET-SXT-CHL	1	0	0	1
AMP-LEX-CRO-CTX-CHL	1	0	0	1
AMP-AMC-FOX-SXT-CHL-AMK	1	0	0	1
AMP-AMC-LEX-FOX-NAL-CIP	1	0	0	1
AMP-LEX-CRO-CTX-NAL-TET-CHL	1	0	0	1

CRO, ceftriaxone; AZM, azithromycin; FOX, cefoxitin; FEP, cefepime; CTX, cefotaxime; AMK, amikacin; GEN, gentamicin; AMC, amoxycillin-clavulanic acid; CIP, ciprofloxacin; LEX, cefalexin; CHL, chloramphenicol; SXT, trimethoprim-sulfamethoxazole; NAL, nalidixic acid; TET, tetracycline; AMP, ampicillin.

**Table 2:** Number of positive samples (%) for each of the antibiotic resistance genes stratified by source ^1^.

ARG	Farm	Wastewater	Transport	Carcass Rinsate	Cecal	Retail Meat	Total
*tetA*	14 (58%)	4 (80%)	3 (100%)	2 (67%)	5 (100%)	11 (100%)	39 (77%)
*tetB*	13 (56%)	3 (60%)	2 (67%)	2 (67%)	5 (100%)	11 (100%)	36 (71%)
*qnrB*	1 (5.6%)	4 (100%)	1 (33%)	1 (17%)	2 (67%)	7 (78%)	16 (37%)
*qnrS*	0 (0%)	0 (0%)	0 (0%)	0 (0%)	0 (0%)	0 (0%)	0 (0%)
*aac(6)-lb-cr*	0 (0%)	0 (0%)	0 (0%)	0 (0%)	1 (33%)	0 (0%)	1 (2%)
*sul1*	7 (64%)	4 (80%)	1 (50%)	1 (50%)	2 (100%)	9 (100%)	24 (80%)
*sul2*	2 (20%)	4 (80%)	0 (0%)	0 (0%)	1 (50%)	4 (44%)	11 (37%)
*sul3*	5 (50%)	3 (60%)	1 (50%)	0 (0%)	1 (50%)	6 (67%)	16 (53%)
*SHV*	8 (62%)	0 (0%)	NT	1 (100%)	NT	2 (50%)	11 (58%)
*CTX-M*	13 (100%)	1 (100%)	NT	1 (100%)	NT	4 (100%)	19 (100%)
*TEM*	0 (0%)	0 (0%)	NT	0 (0%)	NT	1 (25%)	1 (5%)
Per Sample	61 (36%)	23 (58%)	8 (38%)	8 (25%)	17 (68%)	56 (64%)	174 (46%)

^1^ NT, not tested and ARG, antibiotic resistance gene.

## Supplementary Materials

The following are available online at https://www.mdpi.com/2079-6382/9/12/850/s1, Table S1: Number of Isolates per antibiotic susceptibility profile stratified by source.

## Appendix A

**Table A1:** Putative and confirmed *Escherichia coli* across the farm-to-fork continuum.

Sample	Putative *E. coli*	Confirmed *E. coli*
Week 1	41	19
Week 2	40	28
Week 3	40	37
Week 4	39	37
Week 5	50	40
Truck	0	0
Crate	20	19
Carcass rinsate	20	15
Cecal	20	9
Retail meat	62	53
Wastewater	13	9
Total	345	266

**Table A2:** Primers and control strains for each of the resistance genes.

Target	Gene	Primers Sequence 5′–3′	Control Strain ^1^	Reference
*E. coli*	*uidA-F* *uidA-R*	AAAACGGCAAGAAAAAGCAG ACGCGTGGTTAACAGTCTTGCG	*E. coli* ATCC 25922	[52]
Resistance genes	*tetA-F tetA-R*	GTAATTCTGAGCACTGTCGC CTGCCTGGACAACATTGCTT	*Klebsiella pneumonia* strain GCKP12	[53]
	*tetB-F tetB-R*	CTCAGTATTCCAAGCCTTTG ACTCCCCTGAGCTTGAGGGG	*E. coli* strain PN091E1Il	[53]
	*qnrB-F qnrB-R*	GGAATCGAAATTGGCCACTG TTTGCCGTTCGCCAGTCGAA	*K. pneumonia* strain KP224	[31]
	*qnrS-F qnrS-R*	CACTTTGATGTCGCAGAT CAACATACCCAGTGCTT	*K. pneumonia* strain KP230	[31]
	*aac(6)-lb-cr-F aac(6)-lb-cr-R*	GATGCTCTATGGGTGGCTAA GGTCCGTTTGGATCTTGGTGA	*K. pneumonia* strain GCKP12	[31]
	*sul1-F sul1-R*	CTTCGATGAGAGCCGGCGGC GCAAGGCGGAAACCGCGCC	*C. freundii*	[54]
	*sul2-F sul2-R*	TCGTCAACATAACCTCGGACAC GTTGCGTTTGATACCGGCAC	*C. freundii*	[54]
	*sul3-F sul3-R*	GAGCAAGATTTTTGGAATCG CATCTGCAGCTAACCTAGGGCTTTGGA	*C. freundii*	[54]
	*SHV-F SHV-R*	TTAACTCCCTGTTAGCCA GATTTGCTGATTTCGCCC	*K. pneumoniae* (950117510)	[55]
	*CTXM-F CTXM-R*	GGTTAAAAAATCACTGCGTC TTGGTGACGATTTTAGCCGC	*K. pneumoniae* (945169659)	[55]
	*TEM-F TEM-R*	AAAATTCTTGAAGACG TTACCAATGCTTAATCA	*K. pneumoniae* (945169659)	[55]
Clonality	ERIC 1 ERIC 2	ATGTAAGCTCCTGGGGATTCAC AAGTAAGTGACTGGGGTGAGCG	*E. coli* ATCC 25922	[56]

^1^ All non-ATCC strains were inhouse strains that were verified by whole-genome sequencing. Accession numbers are given in brackets.

## References

[1] Graham J.P., Boland J.J., Silbergeld E. (2007). Growth Promoting Antibiotics in Food Animal Production: An Economic Analysis. Public Health Rep..

[2] Mehdi Y., Létourneau-Montminy M.P., Gaucher M.L., Chorfi Y., Gayatri S., Rouissi T., Brar S.K., Côté C., Ramirez A.A., Godbout S. (2018). Use of antibiotics in broiler production: Global impacts and alternatives. Anim. Nutr..

[3] Filho H.C.K., Brito K.C.T., Cavalli L.S., Brito B.G., Méndez-Vilas A. (2015). Avian Pathogenic Escherichia coli (APEC) - an update on the control. The Battle Against Microbial Pathogens: Basic Science, Technological Advances and Educational Programs.

[4] Singer R. (2015). Therapeutics and antibiotic resistance. 19th World Veterinary Poultry Association Congress in South Africa.

[5] Blaak H., Van Hoek A.H.A.M., Hamidjaja R.A., Van Der Plaats R.Q.J., Kerkhof-De Heer L., De Roda Husman A.M., Schets F.M. (2015). Distribution, numbers, and diversity of ESBL-producing E. coli in the poultry farm environment. PLoS ONE.

[6] Von Eugen K., Nordquist R.E., Zeinstra E., van der Staay F.J. (2019). Stocking density affects stress and anxious behavior in the laying hen chick during rearing. Animals.

[7] Gomes A.V.S., Quinteiro-Filho W.M., Ribeiro A., Ferraz-de-Paula V., Pinheiro M.L., Baskeville E., Akamine A.T., Astolfi-Ferreira C.S., Ferreira A.J.P., Palermo-Neto J. (2014). Overcrowding stress decreases macrophage activity and increases Salmonella Enteritidis invasion in broiler chickens. Avian Pathol..

[8] Souillard R., Répérant J.M., Experton C., Huneau-Salaun A., Coton J., Balaine L., Le Bouquin S. (2019). Husbandry practices, health, and welfare status of organic broilers in France. Animals.

[9] Founou L.L., Founou R.C., Essack S.Y. (2016). Antibiotic resistance in the food chain: A developing country-perspective. Front. Microbiol..

[10] Chang Q., Wang W., Regev-Yochay G., Lipsitch M., Hanage W.P. (2015). Antibiotics in agriculture and the risk to human health: How worried should we be?. Evol. Appl..

[11] Antimicrobial Resistance: Tackling a Crisis for the Health and Wealth of Nations. https://amr-review.org/sites/default/files/AMR%20Review%20Paper%20-%20Tackling%20a%20crisis%20for%20the%20health%20and%20wealth%20of%20nations_1.pdf.

[12] Olonitola O.S., Fahrenfeld N., Pruden A. (2015). Antibiotic resistance profiles among mesophilic aerobic bacteria in Nigerian chicken litter and associated antibiotic resistance genes. Poult. Sci..

[13] Han X., Guan X., Zeng H., Li J., Huang X., Wen Y., Zhao Q., Huang X., Yan Q., Huang Y. (2019). Prevalence, antimicrobial resistance profiles and virulence-associated genes of thermophilic Campylobacter spp. isolated from ducks in a Chinese slaughterhouse. Food Control..

[14] Cyoia P.S., Koga V.L., Nishio E.K., Houle S., Dozois C.M., De Brito K.C.T., De Brito B.G., Nakazato G., Kobayashi R.K.T. (2019). Distribution of ExPEC virulence factors, blaCTX-M, fosA3, and mcr-1 in escherichia coli isolated from commercialized chicken carcasses. Front. Microbiol..

[15] Whole-Genome Sequencing for Surveillance of Antimicrobial Resistance. https://www.who.int/publications/i/item/9789240011007.

[16] Mellata M. (2013). Human and Avian Extraintestinal Pathogenic *Escherichia coli*: Infections, Zoonotic Risks, and Antibiotic Resistance Trends. Foodborne Pathog. Dis..

[17] Aarestrup F.M. (2004). Monitoring of antimicrobial resistance in animals: Principles and Limitations. J. Vet. Med..

[18] Yatsuyanagi J., Saito S., Sato H., Miyajima Y., Amano K.I., Enomoto K. (2002). Characterization of enteropathogenic and enteroaggregative Escherichia coli isolated from diarrheal outbreaks. J. Clin. Microbiol..

[19] Theobald S., Etter E.M.C., Gerber D., Abonlik C. (2019). Antibiotic Resistance Trends in Escherichia coli in South African Poultry:2009–2015. Foodborne Pathog. Dis..

[20] Ewers C., Antão E.M., Diehl I., Philipp H.C., Wieler L.H. (2009). Intestine and environment of the chicken as reservoirs for extraintestinal pathogenic Escherichia coli strains with zoonotic potential. Appl. Environ. Microbiol..

[21] Nauta M., Van Der Fels-Klerx I., Havelaar A. (2005). A poultry-processing model for quantitative microbiological risk assessment. Risk Anal..

[22] Kelly L.A., Hartnett E., Gettinby G., Fazil A., Snary E., Wooldridge M. (2003). Microbiological safety of poultry meat: Risk assessment as a way forward. Worlds. Poult. Sci. J..

[23] Oguttu J.W., Veary C.M., Picard J.A. (2008). Antimicrobial drug resistance of Escherichia coli isolated from poultry abattoir workers at risk and broilers on antimicrobials. J. S. Afr. Vet. Assoc..

[24] Johnson T.J., Logue C.M., Johnson J.R., Kuskowski M.A., Sherwood J.S., Barnes H.J., DebRoy C., Wannemuehler Y.M., Obata-Yasuoka M., Spanjaard L. (2012). Associations Between Multidrug Resistance, Plasmid Content, and Virulence Potential Among Extraintestinal Pathogenic and Commensal Escherichia coli from Humans and Poultry. Foodborne Pathog. Dis..

[25] Thibodeau A., Quessy S., Guévremont E., Houde A., Topp E., Diarra M.S., Letellier A. (2008). Antibiotic resistance in Escherichia coli and Enterococcus spp. isolates from commercial broiler chickens receiving growth-promoting doses of bacitracin or virginiamycin. Can. J. Vet. Res..

[26] Winkler M.L., Papp-Wallace K.M., Hujer A.M., Domitrovic T.N., Hujer K.M., Hurless K.N., Tuohy M., Hall G., Bonomo R.A. (2015). Unexpected challenges in treating multidrug-resistant gram-negative bacteria: Resistance to ceftazidime-avibactam in archived isolates of Pseudomonas aeruginosa. Antimicrob. Agents Chemother..

[27] Rychen G., Aquilina G., Azimonti G., Bampidis V., de Lourdes Bastos M., Bories G., Chesson A., Cocconcelli P.S., Flachowsky G., Kolar B. (2017). Safety and efficacy of Sacox® microGranulate (salinomycin sodium) for chickens for fattening and chickens reared for laying. EFSA J..

[28] Diarra M.S., Silversides F.G., Diarrassouba F., Pritchard J., Masson L., Brousseau R., Bonnet C., Delaquis P., Bach S., Skura B.J. (2007). Impact of Feed Supplementation with Antimicrobial Agents on Growth Performance of Broiler Chickens, Clostridium perfringens and Enterococcus Counts, and Antibiotic Resistance Phenotypes and Distribution of Antimicrobial Resistance Determinants in Escherichia coli Isolates. Appl. Environ. Microbiol..

[29] Adelowo O.O., Fagade O.E., Agersø Y. (2014). Antibiotic resistance and resistance genes in Escherichia coli from poultry farms, southwest Nigeria. J. Infect. Dev. Ctries..

[30] Awad A., Arafat N., Elhadidy M. (2016). Genetic elements associated with antimicrobial resistance among avian pathogenic Escherichia coli. Ann. Clin. Microbiol. Antimicrob..

[31] Li L., Wang B., Feng S., Li J., Wu C., Wang Y., Ruan X., Zeng M. (2014). Prevalence and characteristics of extended-spectrum β-lactamase and plasmid-mediated fluoroquinolone resistance genes in Escherichia coli isolated from chickens in Anhui Province, China. PLoS ONE.

[32] Grossman T.H. (2016). Tetracycline antibiotics and resistance. Cold Spring Harb. Perspect. Med..

[33] Dessie H.K., Bae D.H., Lee Y.J. (2013). Characterization of integrons and their cassettes in Escherichia coli and Salmonella isolates from poultry in Korea. Poult. Sci..

[34] Redgrave L.S., Sutton S.B., Webber M.A., Piddock L.J.V. (2014). Fluoroquinolone resistance: Mechanisms, impact on bacteria, and role in evolutionary success. Trends Microbiol..

[35] Hooper D.C., Jacoby G.A. (2015). Mechanisms of drug resistance: Quinolone resistance. Ann. N. Y. Acad. Sci..

[36] Eibach D., Dekker D., Boahen K.G., Akenten C.W., Sarpong N., Campos C.B., Berneking L., Aepfelbacher M., Krumkamp R., Owusu-Dabo E. (2018). Extended-spectrum beta-lactamase-producing Escherichia coli and Klebsiella pneumoniae in local and imported poultry meat in Ghana. Vet. Microbiol..

[37] Niero G., Bortolaia V., Vanni M., Intorre L., Guardabassi L., Piccirillo A. (2018). High diversity of genes and plasmids encoding resistance to third-generation cephalosporins and quinolones in clinical Escherichia coli from commercial poultry flocks in Italy. Vet. Microbiol..

[38] Geornaras I., Hastings J.W., Von Holy A. (2001). Genotypic Analysis of Escherichia coli Strains from Poultry Carcasses and Their Susceptibilities to Antimicrobial Agents. Appl. Environ. Microbiol..

[39] Fair R.J., Tor Y. (2014). Perspectives in Medicinal Chemistry Antibiotics and Bacterial Resistance in the 21st Century. Perspect. Medicin. Chem..

[40] Stokes H.W., Gillings M.R. (2011). Gene flow, mobile genetic elements and the recruitment of antibiotic resistance genes into Gram-negative pathogens. FEMS Microbiol. Rev..

[41] Saleha A., Myaing T.T., Ganapathy K., Zulkifli I., Raha R., Arifah K. (2009). Possible Effect of Antibiotic-Supplemented Feed and Environment on the Occurrence of Multiple Antibiotic Resistant Escherichia coli in Chickens. Int. J. Poult. Sci..

[42] Baron S., Jouy E., Larvor E., Eono F., Bougeard S., Kempf I. (2014). Impact of third-generation-cephalosporin administration in hatcheries on fecal Escherichia coli antimicrobial resistance in broilers and layers. Antimicrob. Agents Chemother..

[43] Molechan C., Amoako D.G., Abia A.L.K., Somboro A.M., Bester L.A., Essack S.Y. (2019). Molecular epidemiology of antibiotic-resistant Enterococcus spp. from the farm-to-fork continuum in intensive poultry production in KwaZulu-Natal, South Africa. Sci. Total Environ..

[44] Pillay S., Amoako D.G., Abia A.L.K., Somboro A.M., Shobo C.O., Perrett K., Bester L.A., Essack S.Y. (2020). Characterisation of Campylobacter spp. isolated from poultry in Kwazulu-Natal, South Africa. Antibiotics.

[45] Langerhuus S.N., Ingvartsen K.L., Bennedsgaard T.W., Røntved C.M. (2013). Gram-typing of mastitis bacteria in milk samples using flow cytometry. J. Dairy Sci..

[46] Moffat J., Chalmers G., Reid-Smith R., Mulvey M.R., Agunos A., Calvert J., Cormier A., Ricker N., Weese J.S., Boerlin P. (2020). Resistance to extended-spectrum cephalosporins in Escherichia coli and other Enterobacterales from Canadian turkeys. PLoS ONE.

[47] Soepranianondo K., Wardhana D.K., Budiarto, Diyantoro (2019). Analysis of bacterial contamination and antibiotic residue of beef meat from city slaughterhouses in East Java Province, Indonesia. Vet. World.

[48] Ebomah K.E., Adefisoye M.A., Okoh A.I. (2018). Pathogenic Escherichia coli Strains Recovered from Selected Aquatic Resources in the Eastern Cape, South Africa, and Its Significance to Public Health. Int. J. Environ. Res. Public Health.

[49] CLSI (2017). Performance Standards for Antimicrobial Susceptibility Testing.

[50] EUCAST Breakpoint Tables for Interpretation of MICs and Zone Diameters. https://www.eucast.org/fileadmin/src/media/PDFs/EUCAST_files/Breakpoint_tables/v_7.1_Breakpoint_Tables.pdf.

[51] WHO Integrated Surveillance of Antimicrobial Resistance in Foodborne Bacteria. https://apps.who.int/iris/bitstream/handle/10665/255747/9789241512411-eng.pdf;jsessionid=9047EE3FB9B6F665B7107EB74BD3E917?sequence=1.

[52] Dungeni M., van der Merwe R., Momba M.N.B. (2010). Abundance of pathogenic bacteria and viral indicators in chlorinated effluents produced by four wastewater treatment plants in the Gauteng Province, South Africa. Water SA.

[53] Sengeløv G., Halling-Sørensen B., Aarestrup F.M. (2003). Susceptibility of Escherichia coli and Enterococcus faecium isolated from pigs and broiler chickens to tetracycline degradation products and distribution of tetracycline resistance determinants in E. coli from food animals. Vet. Microbiol..

[54] Byrne-Bailey K.G., Gaze W.H., Kay P., Boxall A.B.A., Hawkey P.M., Wellington E.M.H. (2009). Prevalence of sulfonamide resistance genes in bacterial isolates from manured agricultural soils and pig slurry in the United Kingdom. Antimicrob. Agents Chemother..

[55] Chirindze L.M., Zimba T.F., Sekyere J.O., Govinden U., Chenia H.Y., Sundsfjord A., Essack S.Y., Simonsen G.S. (2018). Faecal colonization of E. coli and Klebsiella spp. producing extended-spectrum beta-lactamases and plasmid-mediated AmpC in Mozambican university students. BMC Infect. Dis..

[56] Versalovic J., Koeuth T., Lupski J.R. (1991). Distribution of repetitive DNA sequences in eubacteria and application to finerpriting of bacterial genomes. Nucleic Acids Res..

