# Supplementary material for: Molecular Epidemiology of Antibiotic-Resistant Escherichia coli from Farm-to-Fork in Intensive Poultry Production in KwaZulu-Natal, South Africa
Source: Antibiotics (Basel). 2020 Nov 27;9(12):850. doi: 10.3390/antibiotics9120850 (PMC7761107; doi:10.3390/antibiotics9120850)
Supplement: Supplementary file 1 [file antibiotics-09-00850-s001.pdf]

antibiotics

MDPI

Table S1. Number of isolates per antibiogram stratified by source.

Antibiogram	Farm (*n* =1 61)	Transport (*n* = 19)	Abattoir (*n* = 15)	Cecal (*n* = 9)	Retail (*n* = 53)	Wastewater (*n* = 9)	Total (*n* = 266)
No resistance	48	9	3	0	25	2	87
AMP	46	1		1	7	1	56
AZM	1				1		2
CHL	1	1					2
CIP					1		1
GEN			2				2
LEX	2				2		4
NAL	2		4		3		9
SXT	1	1	1		1	1	5
TET	33	4	1				38
AMP-AMK	1						1
AMP-CHL	3						3
AMP-LEX	2		1				3
AMP-NAL	2	1					3
AMP-SXT					1		1
AMP-TET	1						1
LEX-TET	1						1
NAL-TET		1	1	4	4		10
SXT-CHL	5	1			2	1	9
TET-CHL	1						1
TET-GEN	1						1
TET-SXT	1			2	2		5
AMP-LEX-CHL	1						1
AMP-NAL-GEN			1				1
AMP-NAL-TET				1	1		2
AMP-TET-SXT	1			1	1		3
NAL-TET-SXT					2	3	5
AMP-TET-SXT-CHL	2						2
NAL-CIP-TET-SXT					1		1
AMP-CTX-TET-SXT-CHL	1						1
AMP-LEX-CRO-CTX-CHL	1						1
AMP-AMC-FOX-SXT-CHL-AMK	1						1
AMP-AMC-LEX-FOX-NAL-CIP						1	1
AMP-LEX-CRO-CTX-NAL-TET-CHL	1						1
